# Phototherapeutic keratectomy: current approaches and changing trends in a tertiary eye clinic

**DOI:** 10.5935/0004-2749.20230055

**Published:** 2023

**Authors:** Sariye Taskoparan, Selim Genc, Semih Cakmak, Ozum Oztutuncu, Burcin Kepez Yildiz, Yusuf Yildirim

**Affiliations:** 1 Department of Ophthalmology, Beyoglu Eye Training and Research Hospital, University of Health Sciences Turkey, Istanbul, Turkey.

**Keywords:** Photorefractive keratectomy, Corneal opacity, Corneal injuries, Corneal dystrophies, Phototherapy, Ceratectomia fotorrefrativa, Opacidade da córnea, Lesões da córnea, Distrofias da córnea, Fototerapia

## Abstract

**Purpose:**

To examine the efficacy of phototherapeutic keratectomy as a treatment for variable pathologies with anterior corneal opacities and evaluate the distribution of phototherapeutic keratectomy indications over the past 10 years.

**Methods:**

The records of 334 eyes from 276 patients who underwent phototherapeutic keratectomy between March 2010 and 2020 were retrospectively reviewed. Etiologies of the patients who underwent phototherapeutic keratectomy were noted, and their changes were examined. Refractive and visual acuity results before and after the operation were recorded and analyzed according to etiology.

**Results:**

The mean age of the patients was 40.7 ± 16.2 years (range: 19-84). The mean follow-up was 25.5 ± 19.1 months (range: 3-96). Phototherapeutic keratectomy was most frequently applied for corneal stromal dystrophies (44%, 151 eyes from 111 patients), and granular dystrophy was the most common phototherapeutic keratectomy indication among corneal dystrophies. Unlike other indications, there has been an increase in the application of phototherapeutic keratectomy for persistent subepithelial opacities due to adenoviral conjunctivitis in the past 10 years. There was a significant increase in visual acuity in all groups except for the recurrent epithelial defect group (p<0.05). The greatest improvement in visual acuity was detected for stromal dystrophies in the granular dystrophy subgroup.

**Conclusion:**

Despite changing indication trends, phototherapeutic keratectomy remains an effective and reliable treatment for anterior corneal lesions.

## Abstract (pt)

**Objetivo:**

Examinar a eficácia da ceratectomia fo­toterapêutica para o tratamento de patologias variáveis que apresentarem opacidades anteriores da córnea, e avaliar a distribuição das indicações de ceratectomia fototerapêutica nos últimos 10 anos.

**Métodos:**

Foram revisados retrospectivamente os prontuários de 276 pacientes, com 334 olhos tratados com ceratectomia fototerapêutica entre março de 2010 e o ano de 2020. As etiologias dos pacientes submetidos à ceratectomia fototerapêutica foram anotadas e suas alterações foram examinadas. Os resultados refrativos e de acuidade visual antes e após a operação foram registrados e analisados de acordo com a etiologia.

**Resultados:**

A idade média dos pacientes foi de 40,7 ± 16,2 anos (faixa: 19-84). O tempo médio de acompanhamento foi de 25,5 ± 19,1 meses (faixa: 3-96). A ceratectomia fototerapêutica foi aplicada com mais frequência para distrofias estromais corneanas (44%, 151 olhos de 111 pacientes); entre as distrofias corneanas como um todo, a distrofia granular foi a indicação terapêutica mais comum desse procedimento. Ao contrário de outras indicações, nos últimos 10 anos houve um aumento na aplicação de ceratectomia fototerapêutica em casos de opacidade subepitelial persistente causada por conjuntivite adenoviral. Houve um aumento significativo na acuidade visual em todos os grupos, exceto para o grupo com defeito epitelial recorrente (p<0,05). A maior melhora na acuidade visual foi detectada em casos de distrofia estromal, no subgrupo das distrofias granulares.

**Conclusão:**

Apesar das mudanças nas tendências de indicação, a ceratectomia fototerapêutica continua sendo uma abordagem terapêutica eficaz e confiável para tratar lesões da córnea anterior.

## INTRODUCTION

Many corneal pathologies (e.g., corneal scarring, dystrophy, degenerations, band keratopathy, and bullous keratopathy) cause significant visual impairment^(1)^. Superficial anterior stromal diseases can be treated with minimally invasive surgical procedures, including superficial keratectomy, lamellar keratoplasty, and excimer lasers, that is, phototherapeutic keratectomy (PTK)^(2)^. At this point, PTK represents a bridge that links medical and surgical treatments^(3)^.

PTK is an excimer laser-based surgical procedure used to treat many corneal disorders. Specifically, the operation involves a 193-nm argon fluoride excimer laser to treat anterior corneal diseases^(4)^. The 193-nm excimer laser has been used to remove superficial corneal opacity, smooth irregular corneal surfaces, and correct refractive errors since its first clinical application in 1988^(5)^. Using PTK, superficial corneal opacification can be ablated, leaving the underlying clear stroma undisturbed^(6)^.

Corneal dystrophies and scars, both infectious and noninfectious, are the most common causes of potentially reversible visual impairment^(7)^. Besides macular^(7)^, granular^(7)^, lattice^(8)^, and map point fingerprint^(8)^ dystrophies, other indications for which PTK is an effective treatment option include recurrent corneal erosion syndromes^(9,11)^ and stromal scar tissue (e.g., postsurgical scars)^(12)^.

PTK can be used for many refractive and/or therapeutic indications^(3)^. In this study, we aimed to investigate the efficacy of PTK as the treatment for anterior corneal pathologies and assess the refractive and therapeutic effects of PTK. We also aimed to show the change in the indications trend in our clinic over the years.

## METHODS

In this retrospective study, 387 eyes from 303 patients who underwent PTK at University of Health Science Turkey, Beyoglu Eye Training and Research Hospital between March 2010 and 2020 were reviewed. After applying inclusion criteria, a total of 334 eyes from 276 patients with a postsurgical follow-up of at least 3 months were included in the analyses.

After a thorough explanation of PTK, written informed consent was obtained from each participant in accordance with the tenets outlined in the Declaration of Helsinki. Approval was obtained from the Institutional Review Board of the University of Health Science Turkey, Beyoglu Eye Training and Research Hospital, Istanbul, Turkey.

The PTK indication for each eye was noted before the procedure. Treatment data and duration information from the patients who were retreated were recorded. The inclusion criteria were minimum age of 18 years, presence of epithelium-basal membrane dystrophies, and significant deterioration in best-corrected distance visual acuity (CDVA). Cases with preoperative deep central corneal leukoma, a previous history of ocular surgery, pediatric cases (<18 years old), and postoperative follow-up shorter than 3 months were excluded from the analysis.

Detailed anamneses of all patients were recorded before the operation. Patients were assessed preoperatively (baseline) and at 3 months and 1 year postoperatively. Objective refraction measurements using an auto refractometer (KR-1 Auto Kerato-Refractometer, Topcon, Japan) and manifest refraction measurements using the fogging technique were performed at each examination. Moreover, uncorrected (UDVA) and corrected (CDVA) distance visual acuity were recorded. The anterior and posterior segments were evaluated in detail using slit-lamp biomicroscopy. Anterior segment optic coherence tomography (AS-OCT, Visante, Carl Zeiss Meditec, CA, USA) was performed in all cases except for ones with recurrent epithelial defects before surgery.

Surgical Procedure: After topical corneal anesthesia, the patients were treated with Schwind Amaris 750S (Schwind eye-tech-solutions GmbH & Co. KG, Kleinostheim, Germany) excimer laser system with a range of 6.0-8.0 mm ablation zone diameter, depending on lesion size. In recurrent corneal erosions (RCE), epithelial debridement was performed mechanically during the PTK procedure. Contrarily, during the trans-PTK procedure, epithelial debridement was performed using computer-aided production (CAM) mode without mechanical intervention. The total ablation depth in stromal dystrophies and subepithelial deposits was determined by the opacity depth indicated by the AS-OCT measurements performed before the procedure. After ablation, mitomycin-C (MMC, 0.02%) was applied for 30 seconds in all eyes, except for those with recurrent corneal erosions. All patients were treated with therapeutic contact lenses until the epithelial defect healed after the surgery; artificial tears and topical antibiotics were also prescribed. After the epithelial defect healed completely, fluorometholone drops (1%) were added to the treatment regimen four times per day for 1 month and were reduced gradually.

Statistical Analysis: Statistical analysis was performed using the SPSS for Windows software (version 22.0, SPSS, Inc, New York, NY, USA). The normality of all data distributions was confirmed using the Kolmogorov-Smirnov test. Categorical variables were expressed as absolute numbers and percentages. Continuous variables were reported as means with standard deviations (SD) and ranges. Visual acuity before and after the operation was compared with the Wilcoxon test. The changes in visual acuity between the groups were compared using a one-way ANOVA test. A p-value below 0.05 was considered statistically significant.

## RESULTS

The baseline demographic and preoperative data of 334 eyes from 276 patients are given in table 1. The mean age of the patients was 40.7 ± 16.2 years (range: 19-84). The mean follow-up was 25.5 ± 19.1 months (range: 3-96).

**Table 1:** The baseline demographic and preoperative data of the patients

	Mean ± SD	Range
Age (year)	40.7 **±** 16.2	19-84
Female/male	136 / 140	-
Right/left	152 / 182	-
Minimum pachymetry (µm)	492.6 **±** 63.4	300-676
Maximum ablation depth (µm)	72.7 **±** 32.1	10-142
Optik zone (mm)	6.8 **±** 0.43	6-8
Follow-up (month)	25.5 **±** 19.1	3-96

SD= Standard deviations.

**Figure 1:**
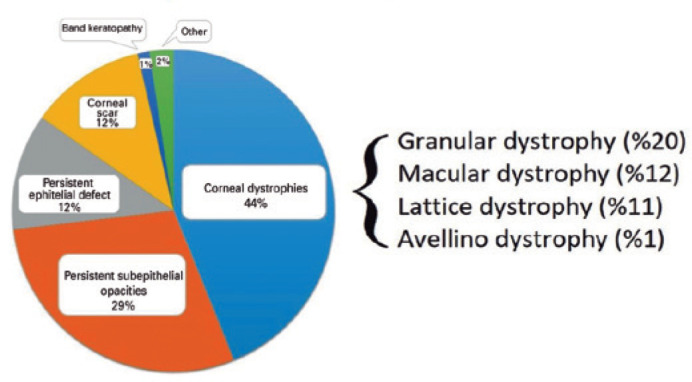
Distribution of indications for phototherapeutic keratectomy.

PTK was most often applied for corneal stromal dystrophies (44%, 151 eyes from 111 patients), and granular dystrophy was the most common PTK indication among corneal dystrophies. The etiological distribution of the cases included in the present study is given in figure 1.

**Figure 2:**
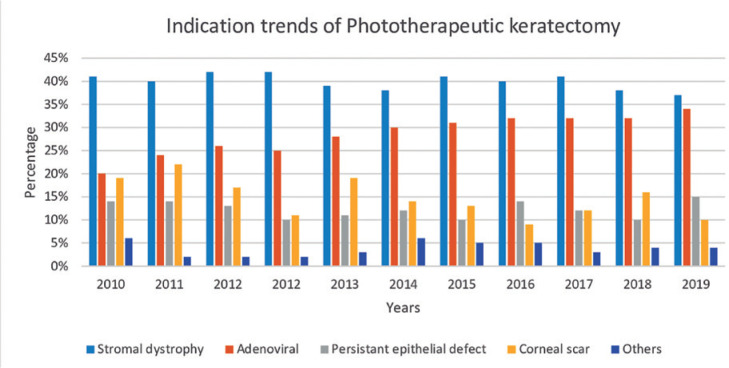
Variable indication trends of phototherapeutic keratectomy.

Unlike other indications, there has been an increase in the application of PTK for persistent subepithelial opacities due to adenoviral conjunctivitis in the past 10 years. Figure 2 shows the changes in the indications for PTK from 2010 to 2019.

**Figure 3:**
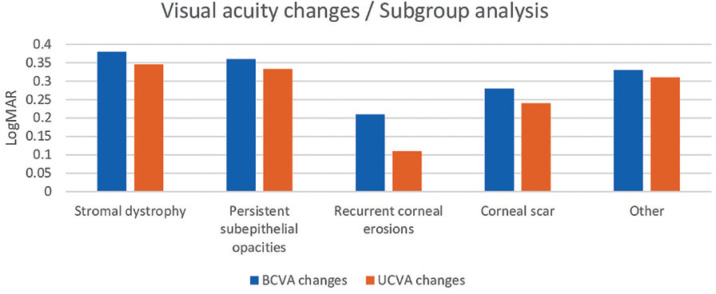
The postoperative change in visual acuity for different indication subgroups.

The preoperative mean spherical equivalent was-0.28 ± 3.13 diopters (D), and the postoperative spherical equivalent was 0.79 ± 3.63 D at the end of the follow-up. The change in visual acuity of all patients before and after the procedure is given in figure 3. There was a significant increase in visual acuity in all groups except for the recurrent epithelial defect group (p<0.05). The greatest improvement in visual acuity was detected for stromal dystrophies in the granular dystrophy subgroup.

Recurrence was the most common in the band keratopathy group (3 eyes, 0.9%). Furthermore, it was observed in one patient with granular dystrophy and two with macular dystrophy. In a persistent subepithelial infiltrate group, no recurrence was observed during the long-term follow-up.

Minimal haze formation was observed in five eyes of five patients (1.5%) at 1 month postoperatively. All cases responded to steroid treatment.

The complications associated with PTK are given in table 2.

**Table 2:** Complications of phototherapeutic keratectomy

	Stromal dystrophy				Persistent subepithelial opacities	Persistent epithelial defect	Band keratopathy	Corneal scar	Other
	Granular dystrophy	Macular dystrophy	Avellino dystrophy	Lattice dystrophy					
Recurrence	1	2	-	-	-	-	3	-	-
Retreatment	-	2	-	-	-	-	1	2	-
Haze	-	1	-	1	2	-	-	1	-
Delayed epithelial healing	-	-	-	1	-	-	-	-	-

## DISCUSSION

Phototherapeutic keratectomy has been an effective excimer laser-based treatment for corneal diseases for ~30 years^(13)^. It is a safe and minimally invasive procedure that can be performed to relieve superficial opacities, such as corneal scars, degeneration, and dystrophies. The indications trend of PTK has changed over the years. In the present study, we evaluated visual rehabilitation and therapeutic efficacy of PTK as the treatment for variable pathologies with anterior corneal opacities and the changing indications trend at our clinic over the past 8 years.

Most often, PTK is used for anterior corneal dystrophies, recurrent corneal erosions, and elevated corneal lesions^(1)^. Symptomatic bullous keratopathy^(14,15)^, pterygium^(16,17)^ band keratopathy^(14,15)^, post-laser *in situ* keratomileusis (LASIK), and photorefractive keratectomy (PRK) complications are among less common indications^(18-20)^. In keratoconus patients, combined transepithelial PTK and conventional PRK simultaneously followed by corneal crosslinking (CXL; Cretan protocol plus) are used to stabilize the cornea and improve visual acuity^(21)^.

Arfaj et al.^(13)^ divided the patients included in their study into group A and group B. They observed that although the most common indication in group A was granular corneal dystrophy, it was a corneal stromal scar due to trauma or an ulcer in group B.

In a study assessing 12 years of PTK experience, the most common indication was a persistent epithelial defect, which is another common indication in corneal dystrophies^(22)^. In our study, PTK was applied to 40 (12%) eyes due to persistent epithelial defects. Besides this, PTK applications for corneal stromal dystrophies were the most common subgroup every year.

Band keratopathy was one of the earliest indications for PTK^(22)^. We still prefer simple ethylenediaminetetraacetic acid (EDTA) treatment for thin subepithelial calcium deposits. Therefore, PTK application was rarely performed for band keratopathy.

The PTK studies in the literature vary according to series and indications; however, no study has yet evaluated the trends in the indications over the years. In a study by Hersh et al.^(23)^, the most frequent indication was corneal scarring, whereas in a study by Hieda et al.^(6)^, granular dystrophy was the most common indication. Our study, by examining the indications change trend over the years, concluded that PTK for subepithelial opacities has increased in recent years.

PTK increases visual acuity in studies investigating PTK performed for different etiologies^(12,24)^. Visual acuity increased in all groups in our study; however, this increa­se was most frequently observed in the granular dystrophy subgroup. The ablation of the anterior stromal opacities along the visual axis may underlie the greater visual increase in these patients.

Most studies report a hyperopic shift after PTK with a mean between 0.87 and 6.4 D^(22,25-26)^. Removal of the central corneal tissue is expected to reduce the corneal curvature, thereby causing a hyperopic shift; however, there are other potential underlying causes. In a large prospective study by Doğru et al.^(2)^, 112 eyes with superficial corneal opacities exhibited a mean hyperopic shift of 3.42 ± 1.15 D (range: 1.00-5.25 D) in all eyes examined 1-year post-PTK^(27)^. In concordance with the literature, the present study found that the mean hyperopic shift was 1.0 D ~2 years post-PTK.

Studies evaluating PTK for RCE management reported only minor refractive changes as these ablations were superficial (5-7 µm) in most cases^(28)^. In our study, we found that the mean hyperopic shift was 4.2 D in cases requiring PTK to manage RCE, and the average ablation depth in these cases was 10 µm.

The delayed healing of epithelial defects, which are generally expected to heal within 1 week, increases the risk of haze and infection^(3)^. An infection should be expected in such diseased corneas, especially when presenting with an epithelial defect and delayed healing. However, no bacterial keratitis or other infections were seen in this study. Prophylactic use of antibiotics with a bandage contact lens likely minimizes the incidence of infection. However, it is crucial to promptly promote epithelialization to avoid postoperative corneal infection. In our study, no delayed epithelial defect was observed except for one patient who had lattice dystrophy.

Furthermore, delayed epithelial defects may increase haze formation^(3)^. No apparent haze formation was observed in our study due to the low incidence of delayed epithelial defects. Minimal haze was observed in five eyes from five patients (1.5%) 1 month postoperatively. All cases responded to steroid treatment. With the careful use of corticosteroids, efforts to minimize inflammation and promote immediate epithelialization help prevent excessive keratocyte activation and haze formation. Again, in our study, the preoperative use of 0.02% MMC might have prevented the development of severe haze. This is supported by published studies in which the usage of MMC prevented the development of haze after PTK^(29)^.

Depending on the underlying pathology, recurrence rates and patterns may vary after excimer laser ablation. Many studies analyzed various dystrophies relapse rates and models^(30)^. Our study observed recurrence in 3 (0.9%) eyes: one with granular dystrophy and two with macular dystrophy. No recurrence was observed in patients with persistent subepithelial opacities.

Although clinical recurrences may not guarantee repetitive treatments, PTK can be repeated if the cornea is sufficiently thick and does not exhibit signs of substantial topographical flattening^(7,30)^. In our study, repetitive treatment was required for 5 eyes (1.5%; Table 2).

According to the records analyzed in the present study, symptomatic improvement was detected in all patients despite recurrences. However, the lack of a questionnaire is an important limitation of our study. Its retrospective design and the absence of long-term follow-up were also limitations that should be addressed in future studies.

Conclusively, despite the changing indications and rising trends in recent years, PTK remains an effective and reliable treatment for anterior corneal lesions. Future studies with longer follow-up are necessary to evaluate the persistence of these improvements.
